# Vaso-active inotropic scores: Clinical value, pros and cons

**DOI:** 10.1016/j.jhlto.2026.100636

**Published:** 2026-08-22

**Authors:** M. Van Hattem, A. Verzelloni Sef, P.G. Guinot, EEC de Waal

**Affiliations:** a Department of Anesthesiology, Leiden University Medical Center, Leiden, Netherlands; b Department of Anaesthesia and Critical Care, Royal Brompton & Harefield Hospitals, Part of Guy’s and St. Thomas’ NHS Foundation Trust, Royal Brompton Hospital London, London, UK; c Departement Anaesthesia and Intensive Care, Centre Hospitalier Universitaire, Dijon, France; d Department of Anesthesiology, University Medical Center, Utrecht, Netherlands

**Keywords:** (vasoactive) inotropic scores, inotropes, systemic vascular resistance, vascular tone, heart failure

## Abstract

**OBJECTIVE:**

Vasoactive-inotropic scores (VIS) are used as predictors of outcome in pediatric and adult patients with cardiogenic shock undergoing cardiac surgery. VIS is calculated by combining the doses of vasoactive and inotropic drugs using weighted formulas. However, similar VIS values may reflect different hemodynamic states such as cardiogenic shock, vasoplegic shock, or mixed shock. This review summarizes the current literature to further evaluate its clinical utility, and its strengths and limitations.

**METHODS:**

A systematic literature search was performed with keywords “vasoactive” and “inotropic score”. We included English-language human studies with full text availability. Extracted data included VIS formulas, publication year, study population, patient characteristics, timing of VIS assessment, cut-off values, and clinical outcomes.

**RESULTS:**

Ninety-two studies evaluating VIS were identified and 16 different inotropic scores were identified. We divided the groups of patients who needed vasoactive and/or inotropic drugs support for vasoplegia, cardiac dysfunction, or mixed shock states. There was a wide variation in the timing of VIS assessment (pre-operative to 72 h after surgery), and in the VIS cut-off values, limiting the comparison between studies. Moreover, orally administered or intravenous bolus medications were not taken into account.

**CONCLUSION:**

VIS is an easy bedside tool that has been shown to be associated with clinical outcomes. However, current formulas do not consider dose-dependent actions of inotropes or vasopressors, other medications used, and the etiology of the support. There is also substantial heterogeneity in the timing of VIS calculation and cut-off values over different patient populations.

## Background

For many years, vasoactive-inotropic scores (VIS) have been used to predict outcomes in cardiogenic shock, septic shock, and after pediatric or adult cardiac surgery. Initially developed in 1995 to quantify inotropic cardiovascular support following arterial switch operations in neonates,^1^ the inotropic score was later used as a measure of illness severity in congenital heart disease^2^, ^3^ and established as predictor of outcomes after cardiac surgery.^4^, ^5^ Although initially limited to inotropic agents (dopamine, dobutamine, and epinephrine), the newer VIS was developed including both inotropic and vasoactive agents. This expanded VIS was investigated later in different patient populations and used as a predictor and related to outcome. In children, higher VIS in the first 48 h after cardiothoracic surgery were related to longer intensive care unit (ICU) length of stay (LOS), increased morbidity and mortality.^6^, ^7^, ^8^ Moreover, high VIS after pediatric cardiac arrest was associated with increased mortality.^9^

In adults, the outcomes related to the VIS were early graft failure after heart transplantation,^10^ and post-heart transplantation mortality.^5^ Moreover, the preoperative VIS independently predict right ventricular failure after left ventricular assist device (LVAD) implantation.^11^ Additionally, the VIS before extracorporeal membrane oxygenation (ECMO) is a major risk factor for mortality in patients with cardiogenic shock.^10^, ^12^ In adolescents, the VIS on the second day after cardiothoracic surgery was predictive for adverse outcomes.^13^

However, it is unclear if a specific VIS has been calculated based on inotropic agents, vasoactive agents, or a combination of these, and it may relate to different patient groups: heart failure patients, vasoplegic or septic patients, or a combination, such as cardiac surgery patients.

Therefore, we aimed to summarize the current literature published on VIS, detailing relevant values of agents used in their calculation, evaluating pros and cons, and examining in more detail the outcomes of these specific patient groups.

## Methods

We performed this review according to the Preferred Reporting Items for Systematic Reviews and Meta-analyses (PRISMA) guidelines. The study protocol was registered in the International Prospective Register for Systemic Reviews (PROSPERO CRD420251026880).

### Data sources and searches

**Figure 1:**
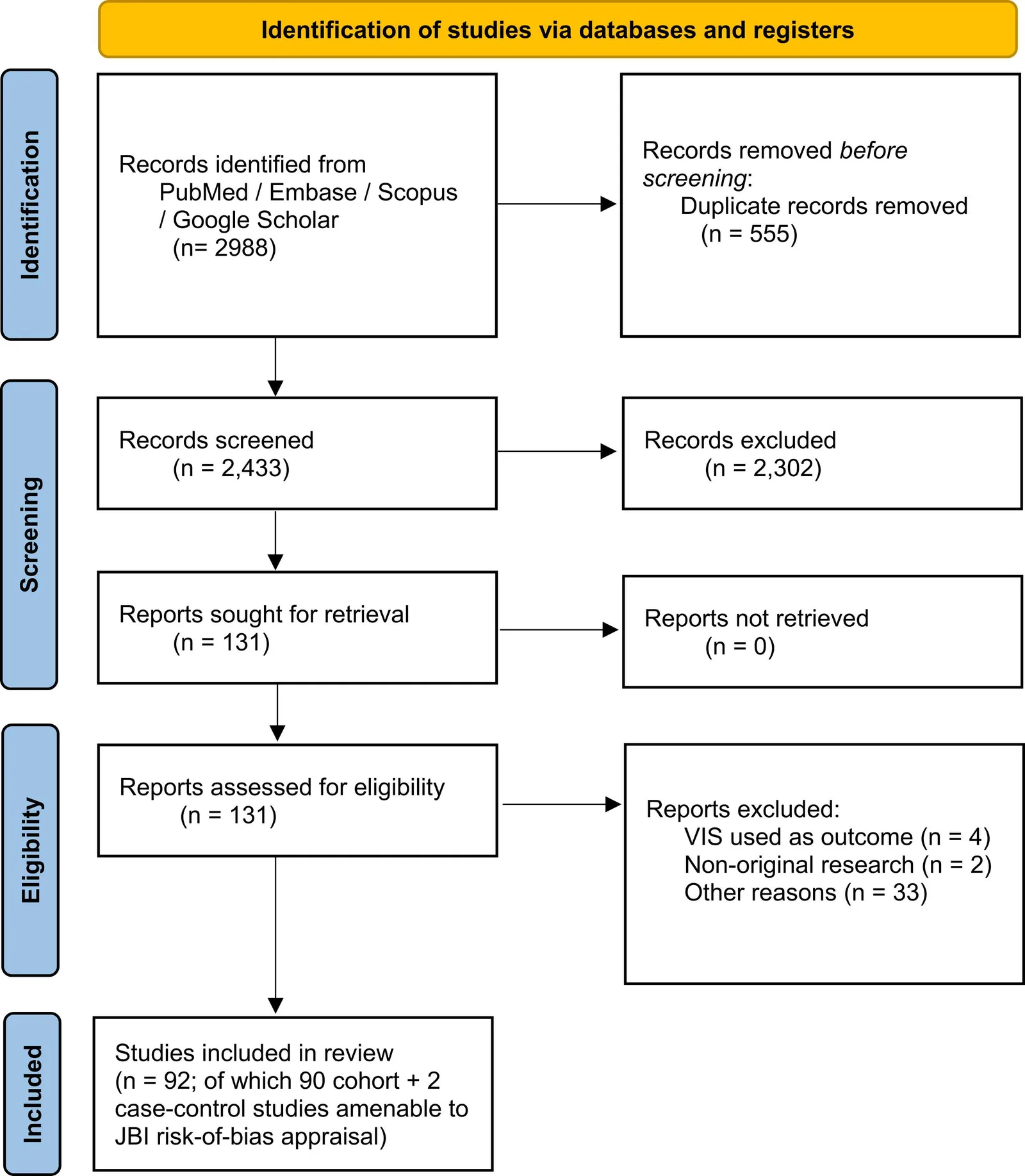
A detailed PRISMA flowchart.

We performed a comprehensive literature search in four databases (Pubmed, Embase, Scopus and Google Scholar) including papers published from January 2015 to March 2026. The MeSH terms Inotropic Score and Vasoactive-Inotropic Score were used. The conducted search is presented in Figure 1.

### Study selection

We included primary studies reporting the Vasoactive-Inotropic Score (VIS) or the Inotropic Score (IS) in human subjects of any age and clinical setting, irrespective of study design. Eligibility required that the VIS or IS are used as a quantitative exposure, predictor, or descriptor of hemodynamic support. Inclusion was restricted to full-text articles published in English. We excluded duplicate publications, conference abstracts, editorials, narrative reviews, case reports involving fewer than 10 patients, and animal studies. After deduplication, two reviewers (MvH, PGG) independently screened titles and abstracts against the eligibility criteria; then independently assessed the full texts of all records retained at the first stage. Disagreements at either stage were resolved by discussion; unresolved disagreements were adjudicated by a third reviewer not involved in the literature search (EdW). Reference lists of all included studies and relevant reviews were hand-searched for additional eligible records, which were then subjected to the same dual-reviewer process.

### Quality assessment

Methodological quality and risk of bias were assessed using the Joanna Briggs Institute (JBI) critical appraisal tools (https://jbi.global/critical-appraisal-tools): the Cohort Checklist (11 items) for the 87 cohort studies, and the Case-Control Checklist (10 items) for the 5 case-control studies. Items addressing length and completeness of follow-up (Cohort Q9–Q10) were rated as not applicable when the primary outcome was an in-hospital event (in-hospital mortality, ICU length of stay, duration of mechanical ventilation, extubation failure, or a composite in-hospital morbidity endpoint), and not applicable responses were excluded from the denominator. Each study was independently appraised by two reviewers (MvH, PGG); disagreements were resolved by discussion and, when needed, adjudicated by a third reviewer (EdW). Overall risk of bias was classified as low (≥70% of applicable items rated “Yes”), moderate (50 – 69%), or high (<50%). Studies were not excluded on the basis of their rating; per-study judgements appear in Supplemental Table S1.

### Data collection

For each included study, we extracted: population (pediatric vs. adult), first author and year of publication, setting, mean age, sample size, primary outcome, VIS or IS formula, VIS timing of measurement (preoperative, intra-operative, peak in the first 24 h, peak in the first 48 h, 24-h time-weighted average, peak postoperative, or other), VIS or IS threshold(s) reported, outcome predicted, effect measure (odds ratio, hazard ratio, AUROC, sensitivity/specificity, or descriptive statistics), and the corresponding 95% confidence interval where available clinical setting and study design.

For the qualitative synthesis, studies were grouped by the dominant clinical phenotype of the cohort into three categories: vasoplegic shock (predominantly septic or distributive etiology), cardiogenic shock (acute heart failure, post-cardiotomy low-output syndrome, or VA-ECMO support), and mixed vasoplegic–cardiogenic shock populations (cardiac surgery cohorts in which both phenotypes coexist by design). Pediatric and adult cohorts were analyzed separately within each category. Two studies of congenital diaphragmatic hernia were grouped within the pediatric cardiac surgery stratum because their perioperative course is dominated by the same cardiovascular and respiratory instability that characterizes the postoperative cardiac-surgery population for which the VIS was developed.^53^, ^91^

### Data synthesis and analysis

Continuous variables are reported as median [interquartile range] or mean (± standard deviation); categorical variables as n (%). The clinical, methodological, and outcome heterogeneity of the included studies (different populations, VIS formulations, timing windows, outcome definitions, and effect metrics) precluded quantitative meta-analysis. The synthesis is therefore qualitative, reported according to PRISMA 2020 and structured by clinical phenotype × age stratum (pediatric versus adult).

For the threshold analysis, within each phenotype × age stratum, the median threshold was computed and accompanied by its 95% confidence interval (CI95%), for strata with n ≤ 5, the interval defaults to the observed range. Threshold distributions are displayed as violin plots with embedded boxplots and individual study points; violin shapes for strata with n ≤ 5 are illustrative only because kernel density estimation is unstable at small sample sizes. All analyses and figures were produced with R version 4.5.2 (R Foundation for Statistical Computing, Vienna, Austria).

## Results

### Study selection

The literature search retrieved 2988 records from PubMed, Embase, Scopus, and Google Scholar. After deduplication, 2433 records were screened based on title and abstract of which 2302 were excluded as off-topic. A total 131 full-text articles were assessed for eligibility. Thirty-nine were excluded for the following reasons: VIS reported only as part of an outcome definition (n = 4), non-original research (n = 2), and other reasons (n = 33), including off-topic articles, duplicate publications, non-English articles, conference abstracts, and animal studies. Ninety-two studies met the inclusion criteria (Figure 1, PRISMA 2020 flow diagram). Of these, 87 were cohort studies eligible for JBI critical appraisal, and 5 were case-control studies assessed using the JBI Case-Control Checklist (Supplemental Table S1).

### Study characteristics

Cohorts were pediatric in 52 studies (56.5%) and adult in 40 studies (43.5%). By clinical phenotype (categories aligned with Table 1): pediatric distributive shock, predominantly septic, including one preterm-neonate cohort 6 (6.5%); pediatric cardiac surgery / cardiac ICU 46 (50.0%); adult shock (septic and non-septic) 6 (6.5%); adult cardiac surgery / mixed (including LVAD, lung transplantation and post-cardiotomy cohorts), 26 (28.3%); adult cardiogenic shock / VA-ECMO 8 (8.7%). Study-level details are summarized in Table 1.

**Table 1:** Summary of published studies and grading in the pediatric and adult population

Study (Author Year)	N	Design	Population	Setting	VIS Formula	VIS Threshold	Primary Outcome	Effect Measure	Timing	GRADE	PMID
**SECTION 1 — PEDIATRIC DISTRIBUTIVE SHOCK (n = 6)**											
Çeleğen 2022^54^	82	Retrospective cohort	P	Septic shock	VIS (Gaies)	≥16.2	Mortality	Assoc. with 6 h lactate clearance	6 h post-admission	⊕◯◯◯ Very Low	36082644
Demirhan 2022^45^	98	Retrospective cohort	P	Late-onset neonatal septic shock	VIS (Gaies)	>20	Mortality	AUC 0.82	VISmax	⊕◯◯◯ Very Low	36399355
Haque 2015^44^	71	Retrospective cohort	P	Septic shock	VIS (Gaies)	>20	In-hospital mortality	100% vs 44% mortality (VIS>20 vs <20)	Highest VIS 0–48 h	⊕◯◯◯ Very Low	25929629
Kallekkattual 2021^20^	176	Retrospective cohort	P	Septic shock	VIS + IS (Gaies/Wernovsky)	>42.5	PICU mortality	AUC 0.88 (0.82–0.92); OR 4.66 (95%CI 1.57–13.87)	Mean VIS every 2 h	⊕◯◯◯ Very Low	34318405
Kharrat 2022^55^	192	Retrospective cohort	P	Preterm neonates <35 wk GA	VIS (Gaies)	≥20	Death or severe neuro injury	OR 4.2–6.7	Peak VIS 24 h	⊕◯◯◯ Very Low	36087459
McIntosh 2017^52^	138	Retrospective cohort	P	Septic shock	VIS (Gaies)	Not defined (continuous)	Ventilator days; ICU LOS	14% increase odds composite/unit VIS at 12 h; r=0.53 for ICU LOS	6 h, 12 h, 24 h, 48 h	⊕◯◯◯ Very Low	28486385
**SECTION 2 — PEDIATRIC CARDIAC SURGERY/ CARDIAC ICU (n = 46)**											
Alam 2018^68^	574	Prospective cohort	P	Surgery for CHD	VIS (Gaies)	>10	Early extubation	VIS>10 independently associated with prolonged MV	After surgery	⊕◯◯◯ Very Low	30333334
Algaze 2017^64^	138	Retrospective cohort	P	Extracardiac Fontan	VIS (Gaies)	Not defined (continuous)	AKI	OR 1.9 (95%CI 1.0–3.4) for stage 2/3 AKI	Day 0 after surgery	⊕◯◯◯ Very Low	27792123
Asfari 2021^82^	121	Retrospective cohort	P	Cardiac surgery	VIS (Gaies)	Not defined (continuous)	Early or late sternal closure	OR 1.1 (95%CI 1.01–1.2) for late sternal closure (p=0.02)	0 h, 12 h, 24 h, 36 h, 60 h	⊕◯◯◯ Very Low	34597200
Bangalore 2017^39^	167	Retrospective cohort	P	Cardiac surgery post-CPB	TIES vs VIS (Gaies formula)	Not defined (continuous — TIES vs VIS)	Death/CPR/ECMO; MV duration	TIES superior to VIS (NS)	H24, H48 post-op	⊕◯◯◯ Very Low	28287056
Beken 2021^81^	199	Retrospective cohort	P	Cardiac surgery	VIS (Gaies)	Not defined (continuous)	AKI	OR 1.02 (95%CI 1.0–1.04) for AKI development (p=0.008)	24 h	⊕◯◯◯ Very Low	33492453
Butts 2012^32^	76	Retrospective cohort (secondary analysis RCT)	P	Cardiac surgery	VIS (Gaies)	15	MV duration	r=0.36 (MV); r=0.27 (ICU LOS); r=0.22 (hospital charges)	36 h	⊕◯◯◯ Very Low	22349666
Conlon 2015^9^	58	Retrospective case series	P	Out-of-Hospital Cardiac Arrest- cardiac ICU	Conlon extended VIS (+Phenylephrine 100×, Vasopressin 10,000×)	Not defined (continuous)	Survival following cardiac arrest	OR 13.7 (95%CI 1.54–122) decreased LVSF on mortality	Within 24 h post-ROSC	⊕◯◯◯ Very Low	25560427
Crow 2014^35^	244	Retrospective cohort	P	Cardiac surgery	VIS (Gaies) + VISindex	Not defined continuous	Composite poor outcome	AUC 0.85 (VISindex) vs 0.80 (maxVIS) for prolonged MVAUC 0.84 (VISindex) vs AUC 0.77 ICU length of stay	0–72 h	⊕◯◯◯ Very Low	24906599
Davidson 2012^8^	70	Prospective cohort	P	CV surgery post-CPB	VIS + IS (Gaies/Wernovsky)	48 h >17	Length of intubation	OR 22.3; OR 8.1; OR 11.3	24 h, 48 h, 72 h	⊕◯◯◯ Very Low	22527067
Deng 2024^90^	510	Retrospective cohort	P	Cardiac surgery <3yrs (RACHS ≥3)	IS (inotropic score, Wernovsky)	Not defined (continuous)	ECMO rescue requirement	HR 4.29; HR 5.10; HR 6.06	Early post-op	⊕◯◯◯ Very Low	38523837
Dilli 2019^73^	119	Prospective cohort	P	Cardiac surgery	VIS (Gaies)	>15.5	MV days; mortality	OR 8.1	72 h	⊕◯◯◯ Very Low	31638004
Friedland-Little 2014^58^	64	Retrospective case-control	P	Norwood operation	VIS (Gaies)	27	Need for ECMO post-op	Peak VIS 27 most prognostic for ECMO need (ROC)	48 h	⊕◯◯◯ Very Low	24100094
Gaies 2010^6^	174	Retrospective cohort	P	Cardiac surgery post-CPB	VIS (Gaies)	24 h >20; 48 h >15	Combined morbidity-mortality	OR 8.1	24 h, 48 h	⊕◯◯◯ Very Low	19794327
Gaies 2014^56^	391	Prospective cohort	P	Cardiac surgery	VIS (Gaies)	>20	Composite poor outcome	OR 6.5 (95%CI 2.9–14.6) composite; OR 13.2 (95%CI 3.7–47.6) mortality	24 h	⊕◯◯◯ Very Low	24777300
Garcia 2016^13^	149	Retrospective cohort	P	Cardiac surgery	VIS (Gaies)	>4.75	Composite adverse outcome	AUC 0.76; OR 1.35	24 h, 48 h	⊕◯◯◯ Very Low	26424215
Hari Gopal 2023^91^	57	Retrospective cohort	P	CDH, neonatal ICU	VIS (Gaies)	Not defined (narrative)	ECMO; in-hospital mortality	VIS ≥7.5 at 6 HOL → ECMO (AUC 0.60); mortality HR 1.10/unit	6–48 h of life; peri-repair	⊕◯◯◯ Very Low	36816370
Kim 2015^59^	126	Retrospective cohort	P	ASO for TGA	VIS (Gaies)	12,5	MV duration; LOS; mortality	OR 1.154 (95%CI 1.024–1.300) mortality; AUC 0.852	Pre-operative	⊕◯◯◯ Very Low	25330856
Kim-Campbell 2020^79^	34	Prospective cohort	P	Cardiac surgery	VIS (Gaies)	Not defined (continuous)	Oxylipins (9-HODE, 13-HODE)	R2=0.25 (9:13-HODE vs VIS 2-24h post-CPB, p<0.01)	2 h, 24 h, 48 h	⊕◯◯◯ Very Low	31305328
Kulyabin 2020a^66^	121	Retrospective cohort	P	Aortic arch reconstruction	VIS (Gaies)	Not defined (continuous)	AKI	OR 1.79 (95%CI 1.33–2.41) early mortality; OR 1.60 (1.35–1.91) AKI	24 h	⊕◯◯◯ Very Low	31835988
Kulyabin 2020b^71^	45	Prospective randomized (pilot)	P	Aortic arch reconstruction	VIS (Gaies) index (AUC-based)	>12	Composite adverse event post-op	VIS index >12 = risk factor for AKI and early mortality	24 h, 48 h, 72 h	⊕◯◯◯ Very Low	32446921
Kumar 2014^57^	208	Retrospective cohort	P	Cardiac surgery	VIS (Gaies)	>10	Post-op variables	NR (associations with morbidity/mortality; no OR in abstract)	48 h	⊕◯◯◯ Very Low	25316975
Kuraim 2018^72^	565	Prospective cohort	P	Cardiac surgery	VIS (Gaies)	Not defined (continuous)	Need for VA-ECMO post-op	OR 1.02 (95%CI 1.06–1.08) for early VA-ECMO (p<0.001)	Post-op day 1	⊕◯◯◯ Very Low	30202528
Lex 2016^63^	1520	Prospective cohort (secondary analysis)	P	Cardiac surgery	VIS (Gaies)	Not defined (continuous)	Fluid overload	OR 1.01 (95%CI 1.005–1.029) for fluid overload >5%	72 h	⊕◯◯◯ Very Low	26914622
Luo 2020^46^	504	Retrospective case-control	P	TPCP surgery post-CPB	VIS (Gaies)	>9 MV (PMV definition)	Prolonged MV	Intraoperative max VIS = independent predictor of PMV	Max VIS intraoperative	⊕◯◯◯ Very Low	31983510
Miletic 2015^36^	222	Retrospective cohort	P	Cardiac surgery	VIS (Gaies) + VVR composite	VIS48 = 9.5; VVR48 = 22.5	Poor outcome	OR 6.18 (VIS) for prolonged intubation (p<0.0001)	0 h, 48 h	⊕◯◯◯ Very Low	25487233
Murin 2020^78^	615	Retrospective cohort	P	Cardiac surgery	VIS (Gaies)	Not defined (continuous)	Mortality and morbidity	VIS independently associated with morbidity (p<0.0001)	24 h	⊕◯◯◯ Very Low	30602177
Odek 2016^61^	99	Prospective cohort	P	Cardiac surgery	VIS (Gaies)	Not defined (continuous)	Early extubation	Lower VIS associated with early extubation (p<0.05)	PACU admission	⊕◯◯◯ Very Low	27272692
Odek 2018^67^	126	Retrospective cohort	P	Cardiac surgery	VIS (Gaies)	Not defined (continuous)	Hyperglycemia	Higher VIS associated with hyperglycemia (p=0.029)	PACU admission	⊕◯◯◯ Very Low	30968624
Parmar 2017^65^	135	Prospective cohort	P	VSD closure	VIS (Gaies)	Not defined (continuous)	Duration of extubation	OR 0.174 (95%CI 0.002–0.062) for delayed extubation (p=0.039)	Intraoperative	⊕◯◯◯ Very Low	28977199
Pérez-Navero 2019^74^	117	Prospective cohort	P	Cardiac surgery	VIS (Gaies)	>15.5	Low cardiac output syndrome	Specificity 92.87% (95%CI 86.75–98.96); NPV 75.59%	2 h, 12 h, 24 h, 48 h	⊕◯◯◯ Very Low	29910113
Raatz 2019^75^	745	Retrospective cohort	P	Cardiac surgery	VIS (Gaies)	Not defined (continuous)	Chylothorax	OR 1.070 (p=0.001) for chylothorax; OR 1.091 (p=0.019) for serous effusion	Day of surgery	⊕◯◯◯ Very Low	31292607
Radbill 2022^84^	1250	Prospective cohort	P	Cardiac surgery	VIS (Gaies)	Not defined (continuous)	Post-operative arrhythmia	OR 1.03 (p=0.04) for post-operative arrhythmia	48 h	⊕◯◯◯ Very Low	34986912
Sanil 2013^7^	51	Retrospective cohort	P	Heart transplantation	VIS (Gaies)	>15	Morbidity and mortality	ICU LOS 30.2 vs 15.9 days (p=0.01); pressor duration 11.4 vs 6.8d (p=0.02)	24 h, 48 h	⊕◯◯◯ Very Low	23773439
Scherer 2016^60^	164	Prospective cohort	P	Cardiac surgery	VVR score (VIS component)	Not defined (continuous score)	Prolonged hospital LOS	AUC 0.93	6 h, 12 h, 24 h, 48 h	⊕◯◯◯ Very Low	27649997
Schroeder 2018^53^	34	Retrospective cohort	P	CDH, post-repair weaning	VIS (Gaies)	NR	Failure of extubation	Higher VIS in extubation failure group (p=0.013, 95%CI [-14/–1.8])	VIS 24 h before extubation	⊕◯◯◯ Very Low	30117219
Shaath 2020^15^	96 (33+63)	Case-control	P	Cardiac surgery post-CPB	IS (simplified formula)	IS > 14	Sternal reopening	Independent predictor	∼H−6 before reopening	⊕◯◯◯ Very Low	31702705
Siehr 2016^62^	32	Retrospective cohort	P	Modified Norwood	VIS (Gaies)	Not defined (continuous)	In-hospital mortality; LOS	NR (VIS used as covariate, no significant independent effect on mortality)	72 h	⊕◯◯◯ Very Low	26825044
Song 2022^85^	282	Retrospective cohort	P	Cardiac surgery — Fontan	IS (inotropic score)	Not defined (continuous — IS)	Prolonged ICU stay (>75th pct)	Independent predictor	Post-Fontan	⊕◯◯◯ Very Low	36002809
SooHoo 2018^69^	95	Retrospective cohort	P	Norwood operation	VIS (Gaies)	Not defined (continuous)	AKI	OR 1.20 (95%CI 1.06–1.35) for fluid-corrected AKI	0 h, 24 h, 48 h	⊕◯◯◯ Very Low	30157730
Sun 2022^33^	401	Retrospective cohort	P	Cardiac surgery	VIS + IS (Gaies/Wernovsky)	Not defined (continuous — VIS48h best predictor)	Prolonged mechanical ventilation	AUC 0.780; OR 1.188	Intra-op, 2 h, 24 h, 48 h	⊕◯◯◯ Very Low	36069143
Tabbutt 2019^76^	8543	Observational analysis	P	Cardiac surgery	VIS (Gaies)	Not defined (continuous predictor)	Mortality	C-statistic 0.92 for mortality model (VIS as component)	2 h after surgery	⊕◯◯◯ Very Low	30489488
Tadros 2020^77^	104	Retrospective cohort	P	Heart transplantation	VIS (Gaies)	>10	Primary graft dysfunction	AUC >0.8 for PGD (cutoff VIS=10); not independent predictor of mortality	24 h, 48 h	⊕◯◯◯ Very Low	32441792
Talwar 2018^70^	215	Retrospective cohort	P	Bidirectional Glenn	VIS + IS (Gaies/Wernovsky)	Not defined (continuous)	Early outcome determinants	NR (Mean VIS 2.77±2.63; not independently associated with mortality)	Mean 12–72 h	⊕◯◯◯ Very Low	33060917
Yokota 2022^83^	70	Retrospective matched cohort	P	Cardiac surgery	VIS (Gaies)	Not defined (continuous)	AKI	OR 4.2 (95%CI 1.1–16) CS-AKI in Williams syndrome; OR 1.47 (1.14–1.9) per VIS unit	1 h, 6 h, 12 h, 24 h	⊕◯◯◯ Very Low	34982759
Zhang 2018^47^	71	Retrospective case-control	P	ALCAPA surgery	VIS (Gaies)	Not defined (continuous)	Prolonged MV; inotropic support	OR 1.08 (95%CI 1.01–1.17) for prolonged MV	Max VIS intraoperative	⊕◯◯◯ Very Low	30293829
Zhang 2020^80^	451	Retrospective cohort	P	Systemic-pulmonary shunt	VIS (Gaies)	>8.5	Shunt failure	OR 1.294	24 h	⊕◯◯◯ Very Low	
**SECTION 3 — ADULT SHOCK (SEPTIC AND NON-SEPTIC) (n = 6)**											
Kara 2019a^92^	102	Retrospective cohort	A	Traumatic brain injury (non-surgical/distributive)	VIS (Gaies)	≥10	Mortality	Mortality 81.1% vs 21.5% (VIS≥10 vs <10, p<0.0001)	Mean VIS 0–48 h	⊕◯◯◯ Very Low	30649831
Kara 2019b^93^	392	Retrospective cohort	A	Septic shock	VIS (Gaies)	>9.75	In-hospital mortality	Mortality meanVIS >10 (OR3.455),	Mean VIS 0–48 h	⊕◯◯◯ Very Low	
Li 2024^40^	4229	Retrospective cohort	A	Septic shock	VIS (Gaies) + NEE score	>15	28-day mortality	AUC 0.802 (nomogram); VIS>15 independently assoc. with 28-day mortality (p=0.001)	VIS 2 h after sepsis onset	⊕◯◯◯ Very Low	39156134
Ning 2023^18^	8887	Retrospective cohort	A	Septic shock (MIMIC-IV)	VIS (Gaies) + VRR (VIS Reduction Rate)	NR	Mortality	HR 1.79 (95%CI 1.44–2.22); HR 2.07 (95%CI 1.61–2.66); HR 1.43 (95%CI 1.28–1.60)	Dynamic (serial)	⊕◯◯◯ Very Low	37082452
Pölkki 2023^94^	8079	Retrospective cohort	A	Mixed adult ICU	VIS (Gaies) vs cvSOFA	NR	ICU outcomes; comparison to cvSOFA	AUC 0.813	VIS at 24 h	⊕◯◯◯ Very Low	37278095
Song 2021^49^	910	Retrospective cohort	A	Septic shock	VIS (Gaies)	>31	30-day mortality	Mortality 70% (VIS>45) vs 17.2% (VIS 0–5); OR gradient (p<0.001)	Max VIS 0–6 h	⊕◯◯◯ Very Low	33572578
**SECTION 4 — ADULT CARDIAC SURGERY / MIXED (n = 26)**											
Barge-Caballero 2015^5^	390	Retrospective cohort	A	Emergency heart transplantation	VIS (Barge-Caballero variant)	>20	Post-transplant mortality	Hazard ratio 1.80 (P<0.001)	Pre-operative	⊕◯◯◯ Very Low	25797676
Baysal 2021^101^	290	Prospective cohort	A	Elective on-pump CABG	VIS (Gaies)	>5.5	Early post-op morbidity/mortality	Sensitivity 90%, Specificity 88% at VIS>5.5 for morbidity/mortality	End of surgery	⊕◯◯◯ Very Low	33577259
Carmona 2020^98^	68	Retrospective cohort	A	LVAD implantation	VIS (Gaies)	Not defined (continuous)	RV dysfunction	Pre-operative VIS associated with RVF (p=0.028); not independent predictor in multivariable	Pre-operative	⊕◯◯◯ Very Low	32395287
Caruso 2024^29^	75	Retrospective multicenter (PSM)	A	Postcardiotomy CS / VA-ECMO	VIS (Gaies)	Not defined (continuous; PSM-integrated)	In-hospital mortality + morbidity	Mortality 56% vs 20% (p<0.001) post-PSM	Pre-ECMO	⊕◯◯◯ Very Low	38182434
Chen 2020^22^	121	Retrospective cohort	A	VA-ECMO post-CABG	VIS (Gaies)	>60	36-month mortality	HR 1.09 (95%CI 1.02–1.16, p=0.011) for 36-month mortality	6 h before ECMO	⊕◯◯◯ Very Low	32529901
Han 2019^96^	418	Retrospective cohort	A	LVAD implantation	VIS (Gaies)	>20	In-hospital mortality	AUC 0.73 (95%CI 0.64–0.82); OR 1.06 (95%CI 1.03–1.09)	Pre- and post-operative	⊕◯◯◯ Very Low	31201088
Han 2021^99^	90	Retrospective cohort	A	Cardiac surgery + AKI	VIS (Gaies)	>19.5/18.9	Risk of death with AKI	AUC 0.796 (95%CI 0.700–0.892); OR 1.147 (95%CI 1.021–1.290) for mortality	Post-op d1; start RRT	⊕◯◯◯ Very Low	34746256
Hou 2021^100^	1935	Retrospective cohort	A	Cardiovascular surgery	VIS (Gaies)	Not defined (continuous — VIS-max)	Post-operative AKI	AUC 0.84; AUC 0.91; OR 1.29	24 h	⊕◯◯◯ Very Low	33798050
Jiang 2022^103^	84	Retrospective cohort	A	Cardiac surgery AKI on CRRT	VIS (Gaies)	Not defined (continuous)	In-hospital mortality	VIS 24 h after CRRT = independent risk factor for in-hospital mortality	24 h; 2 h before/after CRRT	⊕◯◯◯ Very Low	36083528
Keles 2024^89^	221	Retrospective cohort	A	On-pump CABG	VIS (Gaies)	NR	Morbidity and mortality	VISmax 24 h = optimal timepoint	Intraoperative vs VISmax 24 h	⊕◯◯◯ Very Low	38722119
Knight 2022^104^	245	Retrospective cohort	A	Bilateral lung transplantation	VIS (Gaies)	Not defined (continuous)	AKI	Max VIS significant on univariate but not independent in multivariable analysis	Intraoperative	⊕◯◯◯ Very Low	33966025
Koponen 2019^34^	3213	Retrospective cohort	A	Cardiac surgery	VIS (Gaies)	>30	Composite poor outcome	AUC 0.72 (composite); AUC 0.76 (30-day mortality); AUC 0.70 (1-yr mortality)	First 24 h post-surgery	⊕◯◯◯ Very Low	30857599
Kwon 2022^102^	2149	Retrospective cohort	A	OPCABG	VIS (Gaies)	>10.5	1-year mortality	OR 1.02	48 h post-surgery	⊕◯◯◯ Very Low	35896595
Lim 2017^14^	160	Retrospective case-control	A	Cardiac surgery	VIS (Gaies)	Not defined (total VIS used)	NOMI	OR 1.23 (95%CI 1.04–1.44, p=0.011) for NOMI	24 h	⊕◯◯◯ Very Low	28906389
Liu 2018^95^	112	Retrospective cohort	A	Cardiac surgery	VIS (Gaies)	>6	NIV failure	VIS ≥6 = independent predictor of NIV failure	Before commencing NIV	⊕◯◯◯ Very Low	30069328
Liu 2024^88^	250	Retrospective cohort	A	Adult CHD + CABG	VIS (Gaies)	6.5	Prolonged MV	OR 1.301	End of surgery	⊕◯◯◯ Very Low	38748809
Loforte 2021^106^	499	Retrospective cohort	A	Heart transplantation	IS (inotropic score, Wernovsky)	IS > 10	Severe early graft failure	OR 7.3; OR 5.2; OR 8.5	Pre-operative	⊕◯◯◯ Very Low	32768287
Poterucha 2019^21^	243	Retrospective cohort	A	Cardiac surgery (CHD)	VIS (Gaies)	>3	Composite poor outcome	AUC 0.92 (in-hospital mortality); OR 14.2 (95%CI 7.2–28.2) for maxVIS≥3	24 h, 48 h	⊕◯◯◯ Very Low	30451381
Schnackenburg 2025^86^	334	Retrospective cohort	A	Infective endocarditis — valve surgery	VIS (Gaies)	NR	Post-operative outcomes	Predictive value confirmed	Post-operative	⊕◯◯◯ Very Low	41152385
Singh 2022^105^	687	Retrospective cohort	A	OPCABG	VIS (Gaies)	NR	AKI	NR	Pre- and post-operative	⊕◯◯◯ Very Low	—
Sunavsky 2018^97^	447	Retrospective cohort	A	Urgent listing for heart transplantation	VIS (Gaies)	Not defined (continuous)	HU listing failure	OR 2.0; OR 5.1; OR 1.18	At urgent registration	⊕◯◯◯ Very Low	30085128
Tohme 2023^48^	151	Retrospective cohort	A	Heart transplantation	Extended VIS 2020	NR	1-year mortality and morbidity	HR 2.23; HR 2.85	VISmax 24 h	⊕◯◯◯ Very Low	37067499
Vandenberghe 2022^17^	3415	Retrospective cohort	A	Cardiac surgery post-CPB	Time-weighted VIS / VIS-AUC (Gaies, AUC variant)	Not defined (time-integrated VISAUC)	AKI stage =/>2	No independent association with AKI after adjustment (adjusted OR ∼1.0)	Full ICU period (AUC)	⊕◯◯◯ Very Low	35763994
Xiong 2024^50^	325	Retrospective cohort	A	Heart transplantation (single center)	VIS (Gaies)	NR	Day-30 severe adverse outcomes	VISmax nomogram	VISmax post-transplant	⊕◯◯◯ Very Low	39119063
Yamazaki 2018^30^	129	Retrospective cohort	A	Cardiac surgery	VIS (Gaies)	>5.5	Composite poor outcome	OR 4.87; HR 1.62	End of surgery	⊕◯◯◯ Very Low	29332153
Zhao 2024^87^	379	Retrospective cohort	A	Rheumatic heart valve surgery	VIS (Gaies)	16.5	Early extubation ≤8 h	AUC 0.864	VIS at 24 h	⊕◯◯◯ Very Low	38500035
**SECTION 5 — ADULT CARDIOGENIC SHOCK / VA-ECMO (n = 8)**											
Hu 2024^108^	1742	Retrospective cohort	A	CS on VA-ECMO	VIS (Gaies)	>20	In-hospital mortality	AUC 0.70	VIS at 24 h	⊕◯◯◯ Very Low	38505065
Hyun 2021^42^	160	Retrospective cohort	A	CS — VA-ECMO	VIS (Gaies)	≥32 (pre-ECMO)	In-hospital death	Pre-ECMO VIS≥32 = independent predictor	Pre-ECMO cannulation	⊕◯◯◯ Very Low	34759121
Na 2019^16^	493	Retrospective cohort	A	Cardiogenic shock (medical vs ECMO)	VIS (Gaies)	>39	In-hospital mortality	aOR 3.85 (95%CI 1.60–9.22) VIS 39–85; aOR 10.83 (95%CI 4.43–26.43) VIS>85	Highest VIS 0–48 h	⊕◯◯◯ Very Low	29463462
Ning 2026^19^	3170	Retrospective cohort	A	CS (MIMIC-IV + eICU)	Extended VIS 2020 (VRR dynamic concept)	NR	Prognosis (VIS reduction rate)	VRR highly assoc. with prognosis	Dynamic (serial VIS)	⊕◯◯◯ Very Low	41800354
Sandrio 2022^43^	221	Retrospective cohort	A	Cardio-respiratory ECMO (mixed VV vs VVA)	VIS (Gaies)	NR	Survival; cannulation strategy	AUC 0.68	Pre-ECMO	⊕◯◯◯ Very Low	35566516
Tian 2024^41^	222	Retrospective cohort	A	Post-cardiotomy VA-ECMO	VIS (Gaies) + lactate combined model	VIS>24.3 + Lac>6.85 mmol/L	Mortality — 4 risk groups	[NOT IN ABSTRACT — combination model, see full-text]	VIS at 24 h	⊕◯◯◯ Very Low	39259189
Weeks 2023^107^	265	Retrospective cohort	A	VA-ECMO	VIS (Gaies)	>6	30-day survival	VIS >11.4 = lowest survival quartile	VIS at 24 h	⊕◯◯◯ Very Low	37622440
Zhang 2024^51^	126	Retrospective cohort	A	ECMO (mixed)	VIS (Gaies)	8.67 (VIS-mean D2, 7-day mortality)	AKI	AUC 0.77; OR 1.038; OR 1.048	VIS-max Day 1	⊕◯◯◯ Very Low	38324707

Section 1. Pediatric distributive shock (n = 6).

Section 2. Pediatric cardiac surgery / cardiac ICU (n = 46).

Section 3. Adult shock (Septic and non-septic) (n = 6).

Section 4. Adult cardiac surgery / Mixed (n = 26).

Section 5. Adult cardiogenic shock / VA-ECMO (n = 8). Total: 92 included studies (87 cohort + 5 case-control). All studies graded ⊕◯◯◯ Very Low (observational design). A, adult; AKI, acute kidney injury; ALCAPA, anomalous left coronary artery from pulmonary artery; ASO, arterial switch operation; AUC, area under the curve; CABG, coronary artery bypass grafting; CDH, congenital diaphragmatic hernia; CHD, congenital heart disease; CPB, cardiopulmonary bypass; CRRT, continuous renal replacement therapy; CS, cardiogenic shock; ECMO, extracorporeal membrane oxygenation; HOL, hours of life; ICU, intensive care unit; IS, Inotropic Score; LOS, length of stay; LVAD, left ventricular assist device; MV, mechanical ventilation; NIV, non-invasive ventilation; NOMI, non-occlusive mesenteric ischemia; NR, not reported; OPCABG, off-pump CABG; OR, odds ratio; P, pediatric; PACU, post-anesthesia care unit; PICU, pediatric ICU; PSM, propensity-score matching; RACHS, Risk Adjustment for Congenital Heart Surgery; ROSC, return of spontaneous circulation; RRT, renal replacement therapy; RV, right ventricle; TBI, traumatic brain injury; TGA, transposition of great arteries; TPCP, total pulmonary capillary perfusion; VA, veno-arterial; VIS, Vasoactive-Inotropic Score; VRR, VIS Reduction Rate; VSD, ventricular septal defect.

### Risk of bias

The JBI critical appraisal checklists were applied across the 92 included studies (87 cohort, case-control), with Q9 and Q10 (length and completeness of follow-up) rated *not applicable* when the primary outcome was an in-hospital event. Twenty-four studies (26.1%) were rated low risk of bias, sixty-five (70.7%) moderate risk of bias, and three (3.3%) high risk of bias. Two of the three high-risk of bias studies were case-control designs without independent control selection (,^14^, ^15^); the third was a retrospective cohort with limited adjustment for confounders (^16^). Per-study domain-level judgements appear in Table 1. No study was excluded on the basis of risk of bias.

### Definition of the VIS

Among the 92 included studies, 16 distinct VIS or IS formulations were used (Table 2). The Wernovsky inotrope score combined dopamine, dobutamine, and epinephrine.^1^ The Gaies extension added milrinone, vasopressin, and norepinephrine.^6^ Subsequent variants incorporated phosphodiesterase inhibitors, isoprenaline, levosimendan, phenylephrine, norepinephrine, and vasopressin in heterogeneous weighted ratios. The Barge-Caballero variant^5^ uses different coefficients for dobutamine, dopamine, and vasopressin, which prevents direct numerical comparison of thresholds with cohorts that used the Gaies formulation. Newer formulations include a time-weighted VIS / VIS-AUC^17^ and a dynamic VIS-reduction-rate concept.^18^, ^19^

**Table 2:** Vasoactive-Inotropic Scores formulations identified across the 92 included studies. (16 distinct formulations)

Formulation	Components and weighting	First reference
1. Wernovsky Inotrope Score (IS)	Dopamine + Dobutamine + 100×Epinephrine (µg/kg/min)	Wernovsky 1995^1^
2. Gaies VIS	IS + 10×Milrinone + 10,000×Vasopressin + 100×Norepinephrine	Gaies 2010^6^
3. Conlon extended VIS	Gaies VIS + 100×Phenylephrine + 10,000×Vasopressin	Conlon 2015^9^
4. Barge-Caballero variant	Modified weights for Dobutamine, Dopamine, Vasopressin	Barge-Caballero 2015^5^
5. VIS + Levosimendan (LVIS)	Gaies VIS + 50×Levosimendan	Favia 2013^38^
6. VIS index (Crow)	Cumulative AUC of max VIS over 0–72 h	Crow 2014^35^
7. Vasoactive-Ventilation-Renal (VVR) score	VIS + ventilation index + creatinine ratio	Miletic 2015^36^
8. TIES (Total Inotropic Equivalent Score)	Equivalent-dose conversion of all vasoactives to norepinephrine	Bangalore 2017^39^
9. Norepinephrine-Equivalent (NEE) score	Catecholamine + non-catecholamine vasopressors as norepinephrine equivalents	Li 2024^40^
10. Time-weighted VIS / VIS-AUC	Continuous VIS integrated over ICU stay	Vandenberghe 2022^17^
11. VIS Reduction Rate (VRR)	(VIS_baseline − VIS_t) / VIS_baseline	Ning 2023^18^
12. Extended VIS 2020	Gaies VIS + Methylene blue + Terlipressin + Angiotensin II (proposed)	Belletti 2021^37^
13. VIS + Lactate combined model	VIS + serum lactate, 4-risk-group stratification	Tian 2024^41^
14. Pre-ECMO VIS	Pre-cannulation VIS for VA-ECMO triage	Hyun 2021^42^; Sandrio 2022^43^
15. Maximum VIS (VISmax)	Highest VIS in defined window (variable across studies)	Na 2019,^16^ Hague 2015,^44^ Demirhan 2022,^45^ Luo 2020,^46^ Zhang 2018,^47^ Tohme 2023,^48^ Song 2021,^49^ Xiong 2024,^50^ Zhang 2024^51^
16. Simplified IS (no vasopressin/milrinone)	Dopamine + Dobutamine + 100×Epinephrine, no vasopressors	Shaath 2020^15^

AUC, area under the curve; ECMO, extracorporeal membrane oxygenation; IS, Inotropic Score; NEE, Norepinephrine-Equivalent; TIES (Total Inotropic Equivalent Score); VA, veno-arterial; VIS, Vasoactive-Inotropic Score; VRR, VIS Reduction Rate; VVR Vasoactive-Ventilation-Renal.

### Populations studied and cut-off values

**Figure 2:**
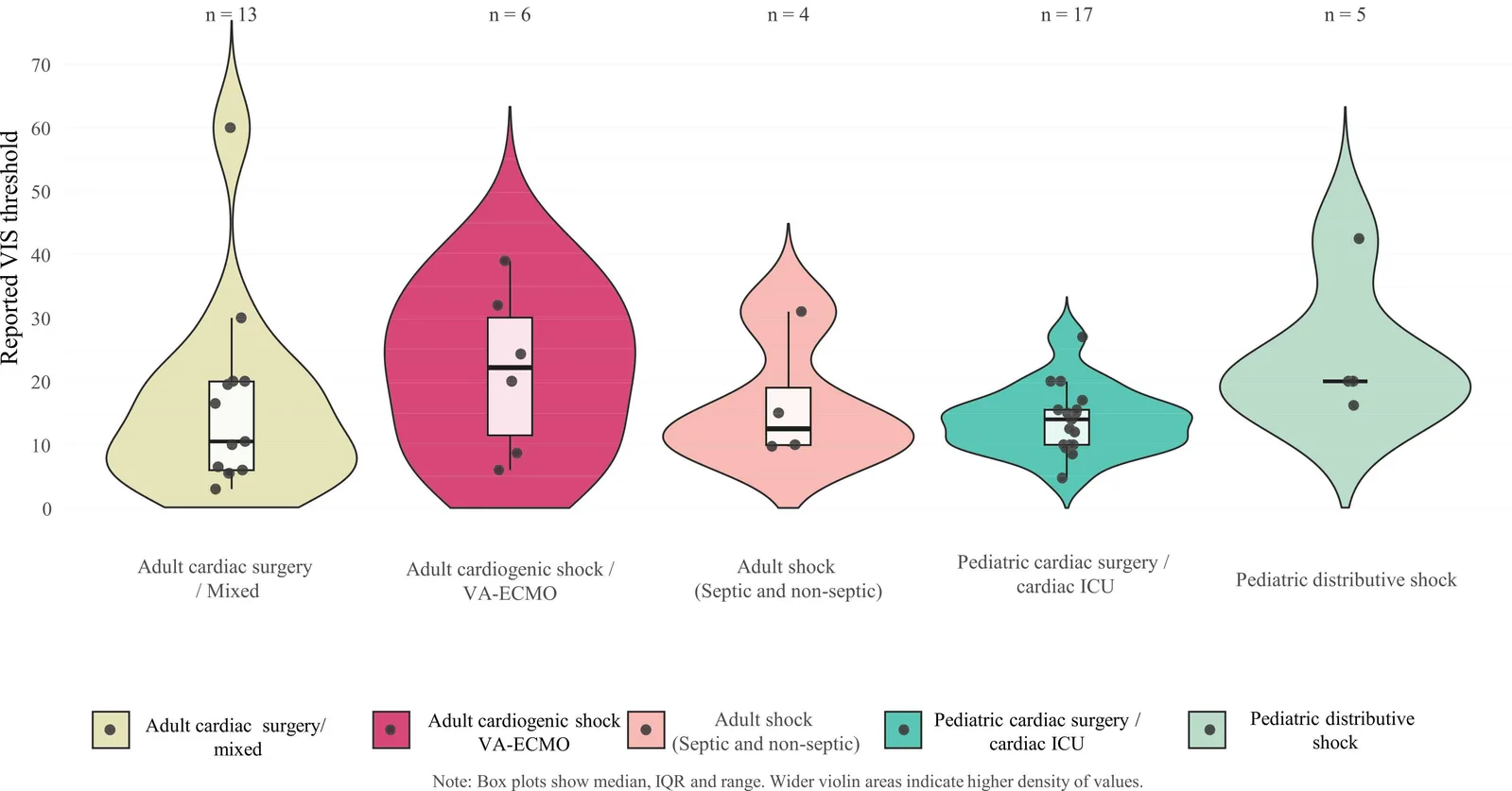
Distribution of reported VIS thresholds by population and clinical context. Violin = density; box = median and IQR; dots = individual studies. n = 45 of 92 studies report a discrete threshold.

**Figure 3:**
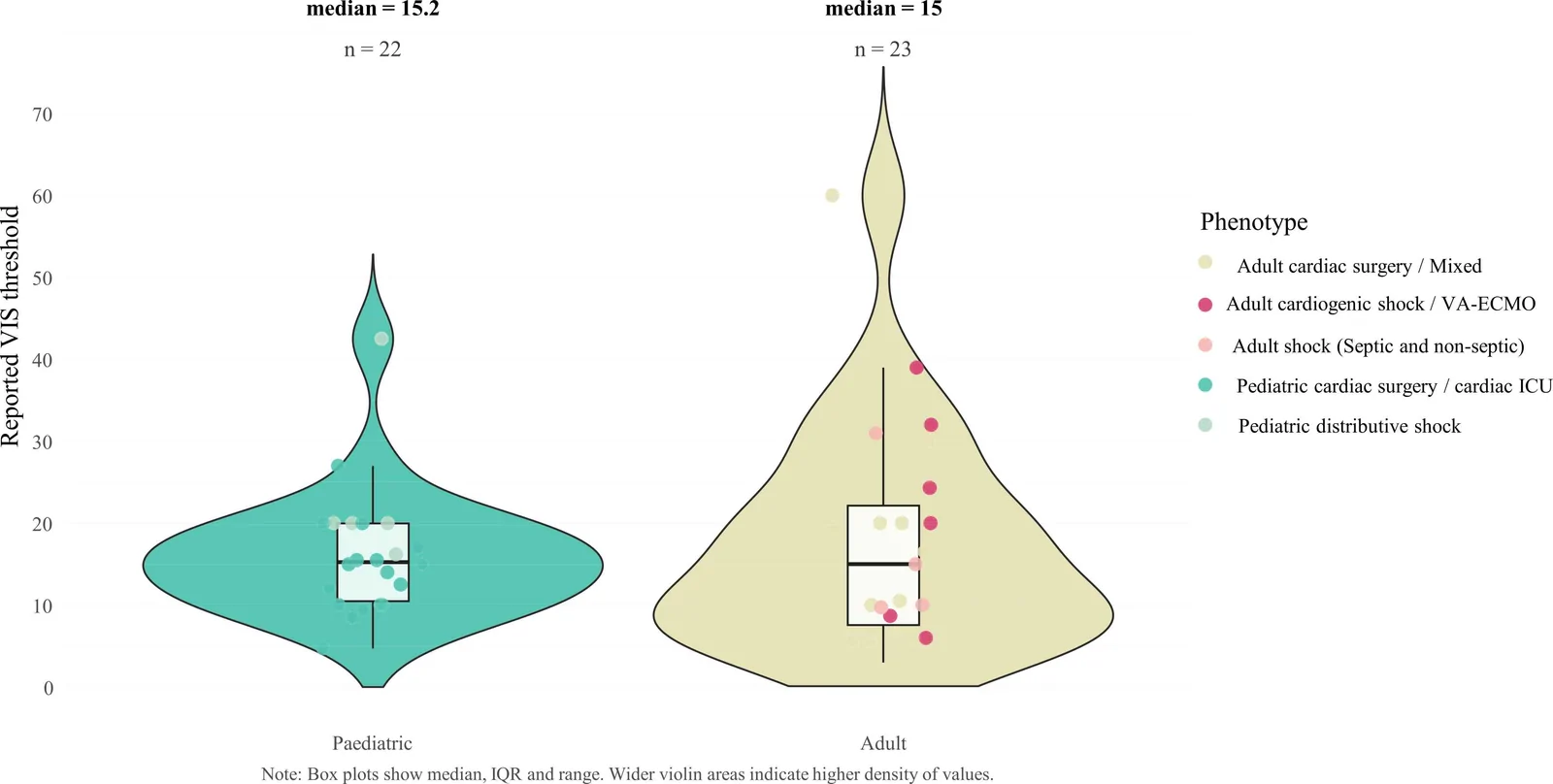
Distribution of reported VIS thresholds by age group. Violin = density; box = median and IQR; dots = individual studies colored by phenotype.

Table 1 lists all included studies by phenotype with the corresponding setting, mean age, sample size, primary outcome, VIS timing, and cut-off. The mixed category corresponds predominantly to cardiac surgery cohorts. The distribution of reported threshold values is plotted by population and clinical context in Figure 2 and stratified by age groups in Figure 3.

A VIS threshold was reported in 45 of 92 studies (48.9%); the remaining 47 studies analyzed VIS as a continuous predictor. Among studies reporting a threshold, the median value was 20 (95% CI 16.2–42.5; n = 5/6) in pediatric distributive shock, 14 (95% CI 10–15.5; n = 17/46) in pediatric cardiac surgery / cardiac ICU, 10.5 (95% CI 5.5–20; n = 13/26) in adult cardiac surgery / mixed, 22.15 (95% CI 6–39; n = 6/8) in adult cardiogenic shock / VA-ECMO, and 12.5 (95% CI 9.75–31; n = 4/6) in adult shock (septic and non-septic) (Table 3). Pediatric thresholds spanned 4.75 (cardiac surgery^13^) to 42.5 (septic shock^20^); adult thresholds spanned 3 (congenital cardiac surgery^21^) to 60 (,^22^ VA-ECMO post-CABG).

**Table 3:** Median VIS threshold by phenotype x age stratum (95% CI, binomial rank-based)

**Stratum**	**n with discrete threshold**	**n total**	**Median VIS**	**95% CI**	**Range**
Pediatric distributive shock	5	6	20	16.2 – 42.5	16.2 – 42.5
Pediatric cardiac surgery / cardiac ICU	17	46	14	10 – 15.5	4.75 – 27
Adult shock (Septic and non-septic)	4	6	12.5	9.75 – 31	9.75 – 31
Adult cardiac surgery / Mixed	13	26	10.5	5.5 – 20	3 – 60
Adult cardiogenic shock / VA-ECMO	6	8	22.15	6 – 39	6 – 39
**All strata**	**45**	**92**	—	—	3 – 60

### Timing of measurement

VIS was measured at heterogeneous time points, ranging from preoperative baseline to 72 h postoperatively. The most frequent windows were peak VIS in the first 24 h, peak VIS in the first 48 h, and a 24-h time-weighted average. Two studies used a dynamic, serial-measurement approach (VIS reduction rate^18^, ^19^).

### Outcomes

Reported primary outcomes were heterogeneous and study-specific (Table 1). Higher VIS values were associated with mortality up to one-year, prolonged mechanical ventilation, longer ICU and hospital length of stay, post-operative ECMO requirement, arrhythmia, non-invasive ventilation failure, right ventricular failure, and acute kidney injury. Effect estimates were reported in heterogeneous metrics (odds ratios, hazard ratios, area under the receiver-operating-characteristic curve), which precluded pooled estimation.

## Discussion

In this review, we investigated the use of VIS, the various scores utilized in clinical practice, and the relation of VIS scores to outcomes in specific settings. High VIS in both children and adults were related to poor outcomes in all groups. However, we observed a large discrepancy between VIS used, their cut-off values and the timing of use in clinical practice. Therefore, we can consider that the generalizability of VIS to the general practice is poor and pros and cons for the use of VIS have to be discussed.

### Pros

Over the last three decades, substantial research has focused on quantification of vasoactive and inotropic support in surgical and ICU settings to better characterize illness severity, predict outcomes, anticipate medical course, and standardize research across studies.

The VIS has emerged out of a need to quantify the magnitude of hemodynamic support in clinical practice as well as research. Many different vasoactive and inotropic drugs are used to calculate VIS depending on the expert experience and/or local practice. VIS is an inexpensive bedside tool-parameter that can be calculated rapidly in perioperative and critical care settings to anticipate the severity of illness of patients, evaluate the progress of a single patient over time, estimate prognosis, enable meaningful discussions with patients and families, and compare patients within similar clinical populations (e.g. heart transplantation). Moreover, VIS is available immediately at the bedside, before laboratory results are available. It has been incorporated in expert consensus documents and clinical guidelines, such as the International Society of Heart and Lung Transplantation (ISHLT) consensus definition of primary graft dysfunction (PGD)^109^). VIS is helpful to objectively compare the amount of hemodynamic support within patient groups and between studies, and as a predictor of outcome in both adults and children, with higher VIS values associated with increased morbidity and mortality (Table 1).

A practical example is the use of VIS in cardiogenic shock to guide early VA-ECMO support decisions, as it predicts outcomes, particularly mortality. A recent study assessed outcomes in patients with post-cardiotomy shock supported by VA-ECMO or conventional therapy according to VIS. When populations were matched on comorbidities and VIS, VA-ECMO remained associated with worse outcomes.^29^

### Cons

VIS is calculated by weighting individual inotropic and vasoactive agents according to their relative potency; however, several important limitations and considerations should be recognized.

First, inotropic agents exert different vasoactive actions due to their differences in α_1_, α_2_, β_1_ and β_2_ receptor affinity^23^ and differences in dose-dependent effects on these receptors resulting in chronotropic effects, inotropic effects and/or vasoactive effects (vasoconstriction versus vasodilation). It does not consider relative interactions between vasoactive and inotropic agents and the use of oral vasoactive medications with their respective half-life. Second, there is no literature supporting the absolute ratios or weights of the various medications used in the calculation of VIS, and the latest formulas are mainly influenced by the weight of vasopressors relative to inotropic support. For instance, high VIS values are primarily due to high vasopressor requirements, which may explain why higher scores were observed in mixed vasoplegic-cardiogenic shock or septic shock patients. Third, both the type and dose of inotropes used, as well as the specific type of shock, may also significantly alter the VIS score. For example, dopamine administration results in a significant decrease in systemic vascular resistance (SVR) in patients with cardiogenic shock, while in patients with septic shock, no significant difference in SVR occurs, all depending on dosages used.^24^, ^25^ The existing inotropic scores do not take into consideration these dose-dependent vasoconstrictive and/or vasodilatory actions of inotropic drugs affecting vasomotor tone and, subsequently, SVR and cardiac output (CO). Moreover, it is known that vasopressin has both vasoconstrictive (V1a) and vasorelaxation (V2) effects, influencing the relative vasopressive effects depending on the distribution of these receptors.^26^ Fourth, the current VIS does not distinguish between the etiology and the need of support. Post-cardiac surgery hypotension for example can be a result of vasoplegia or diminished contractility. Both require a different approach: vasoplegia would be more logically treated with vasopressors, while a patient with diminished cardiac contractility would better benefit from inotropes. But currently there is a trend toward lower use of inotropes and higher rates of vasopressor use while the underlying rate of cardiovascular alteration may not have changed.^27^ As norepinephrine may exert some inotropic effects, treating cardiac inotropic insufficiency with norepinephrine may be associated with a higher VIS score than dobutamine treatment.^28^ It would be more useful in daily practice to discriminate between these groups.

Furthermore, VIS has been developed across different populations with several formulas, we encounter various VIS cut-off values that likely reflect population-specific physiological concerns. A striking age-related difference exists in VIS. Neonates typically require substantially higher VIS values than older children, adolescents, and adults, reflecting their greater need for hemodynamic support and differences in cardiovascular physiology. This was described earlier by Garcia et al. who postulated that interventions in adolescents are less complex and demonstrate better myocardial response to contractility enhancement compared to neonates.^13^ Yamazaki^30^ found also an unexpectedly low VIS cut-off in the adult population compared to the neonatal population because infants and children with congenital heart disease are known to have severely reduced beta-adrenoceptor densities due to downregulation induced by elevated sympathetic tone.^31^ This all demonstrates the clinical heterogeneity of studied patient populations, the variability in timing of VIS assessment, and the wide range of calculated VIS and thresholds of VIS across pediatric and adult cohorts (higher in children compared to adults).

In addition, several studies have reported no significant association between VIS and clinical outcomes, likely reflecting differences in patient complexity, underlying disease processes, and variability in clinical management. In a neonatal study, maximum VIS was not associated with hospital LOS likely due to non-hemodynamic complications unique to this population such as feeding difficulties,^32^ vocal cord paralysis, phrenic nerve injury, and chylothorax.^8^

There is a discrepancy in the timing of VIS, which may be calculated pre-operatively, during surgery, 2 h, 24 h, 48 h, and/or 72 h after surgery. The optimal timing is unclear. Due to these variations in timing of assessment, the comparison of VIS reported in studies is difficult. In a retrospective study comparing the VIS at 24 h and 48 h, the 48 h VIS had a better discriminative power for poor prognosis.^33^ Some studies suggested that a VIS max (calculated as a sum of the maximum dosages of all used inotropes and vasoconstrictors during the first 24 h) is more accurate.^34^ To address this limitation, another study suggested the use of the VIS index. The VIS index is a derivative of the original VIS that incorporates the duration and magnitude of vasopressor/inotropic support using the max VIS every 24 h. A cumulative max VIS in the first 72 h is calculated and corrected by a factor of 2 in the last 24 h period if patients were still using inotropic support to account for prolonged hemodynamic support. The VIS index was significantly associated with poor outcome.^35^ Furthermore, the vasoactive ventilation renal score (VVR-score) incorporates postoperative respiratory, cardiovascular, and renal function, and has been shown to be a more robust predictor of outcome following pediatric cardiac surgery compared to VIS.^36^

The VIS is related to many different poor outcomes (Table 2), but it is more complex than the administered medications. Different from the recent SCAI classification which integrates both medical and mechanical support to define cardiogenic shock, the VIS only focuses on the pharmacological part of the equation. A patient may not have a high need for inotropic or vasopressor support because of the use of temporary or durable mechanical circulatory support (MCS).

Furthermore, although the VIS was originally incorporated in the definition for PGD after heart transplantation,^109^ the recent ISHLT 10-year consensus update on graft dysfunction within the first 72 h after transplantation noted that its applicability for grading PGD may be limited in contemporary clinical practice.^110^

Finally, current VIS formulas do not incorporate newer medications. Belletti et al. proposed a new inotropic score, which includes methylene blue, terlipressin, and angiotensin II.^37^ As additional vasoactive therapies continue to emerge, VIS formulas will require ongoing revision, further limiting the consistency and comparability of the score across studies and over time.

## Conclusion

The inotropic score is a simple, inexpensive bedside tool, and higher VIS values have been shown to be associated with worse clinical outcomes. However, some of the more important counterarguments are that these scores do not consider dose-dependent actions of inotropes and vasopressors, other medications used, and do not discriminate between the etiology of the support. There is a large variability in calculations of VIS, and an important heterogeneity in the timing of VIS calculations cut-off values over different patient populations.

## Declaration of Competing Interest

PGG received fees for lectures from Baxter, AOP, Medtronic, Edwards and Vygon. PGG is consultant for Abbott. EECdW received honoraria for lectures and research grants from Abbott, Cytosorbents and SmartQare. All other authors have no disclosures to report.

## Footnotes

Appendix A Supplemental data associated with this article can be found in the online version at doi:10.1016/j.jhlto.2026.100636.
