# Supplementary material for: Vaso-active inotropic scores: Clinical value, pros and cons
Source: JHLT Open. 2026 Aug 22;14:100636. doi: 10.1016/j.jhlto.2026.100636 (PMC13572079; doi:10.1016/j.jhlto.2026.100636)
Supplement: Supplementary file 1 [file mmc1.docx]

## **Supplementary Table S1.** JBI Critical Appraisal Checklist for Cohort Studies. *Y = Yes | N = No | U = Unclear | NA = Not Applicable | Low ≥ 8 Y | Moderate 5–7 Y | High ≤ 4 Y*

| **Study** | **N** | **Design** | **Q1** | **Q2** | **Q3** | **Q4** | **Q5** | **Q6** | **Q7** | **Q8** | **Q9** | **Q10** | **Q11** | **Y** | **Overall** |
| --- | --- | --- | --- | --- | --- | --- | --- | --- | --- | --- | --- | --- | --- | --- | --- |
| **SECTION 1 — PEDIATRIC DISTRIBUTIVE SHOCK (n = 6)** | | | | | | | | | | | | | | | |
| Çeleğen 2022  [54] | 82 | Retrospective cohort | Y | Y | Y | U | U | Y | Y | Y | U | U | Y | 7 | **Moderate** |
| Demirhan 2022  [45] | 98 | Retrospective cohort | Y | Y | Y | U | U | Y | Y | Y | U | U | Y | 7 | **Moderate** |
| Haque 2015  [44] | 71 | Retrospective cohort | Y | Y | Y | U | U | Y | Y | Y | U | U | Y | 7 | **Moderate** |
| Kallekkattual 2021  [20] | 176 | Retrospective cohort | Y | Y | Y | U | U | Y | Y | Y | U | U | Y | 7 | **Moderate** |
| Kharrat 2022  [55] | 192 | Retrospective cohort | Y | Y | Y | U | U | Y | Y | Y | U | U | Y | 7 | **Moderate** |
| McIntosh 2017  [52] | 138 | Retrospective cohort | Y | Y | Y | U | U | Y | Y | Y | U | U | Y | 7 | **Moderate** |
| **SECTION 2 — PEDIATRIC CARDIAC SURGERY/ CARDIAC ICU (n = 46)** | | | | | | | | | | | | | | | |
| Alam 2018  [68] | 574 | Prospective cohort | Y | Y | Y | U | U | Y | Y | Y | U | U | Y | 7 | **Moderate** |
| Algaze 2017  [64] | 138 | Retrospective cohort | Y | Y | Y | U | U | Y | Y | Y | U | U | Y | 7 | **Moderate** |
| Asfari 2021  [82] | 121 | Retrospective cohort | Y | Y | Y | U | U | Y | Y | Y | U | U | Y | 7 | **Moderate** |
| Bangalore 2017  [39] | 167 | Retrospective cohort | Y | Y | Y | U | U | Y | Y | Y | U | U | Y | 7 | **Moderate** |
| Beken 2021  [81] | 199 | Retrospective cohort | Y | Y | Y | U | U | Y | Y | Y | U | U | Y | 7 | **Moderate** |
| Butts 2012  [32] | 76 | Retrospective cohort (secondary analysis RCT) | Y | Y | Y | U | U | Y | Y | Y | U | U | Y | 7 | **Moderate** |
| Conlon 2015  [9] | 58 | Retrospective case series | Y | Y | Y | U | U | Y | Y | Y | U | U | Y | 7 | **Moderate** |
| Crow 2014  [35] | 244 | Retrospective cohort | Y | Y | Y | Y | Y | Y | Y | Y | U | U | Y | 9 | **Low** |
| Davidson 2012  [8] | 70 | Prospective cohort | Y | Y | Y | Y | Y | Y | Y | Y | U | U | Y | 9 | **Low** |
| Deng 2024  [90] | 510 | Retrospective cohort | Y | Y | Y | U | U | Y | Y | Y | U | U | Y | 7 | **Moderate** |
| Dilli 2019  [73] | 119 | Prospective cohort | Y | Y | Y | U | U | Y | Y | Y | U | U | Y | 7 | **Moderate** |
| Friedland-Little 2014 [58] | 64 | Retrospective case control | Y | Y | Y | U | U | Y | Y | Y | U | U | Y | 7 | **Moderate** |
| Gaies 2010  [6] | 174 | Retrospective cohort | Y | Y | Y | Y | Y | Y | Y | Y | U | U | Y | 9 | **Low** |
| Gaies 2014  [56] | 391 | Prospective cohort | Y | Y | Y | Y | Y | Y | Y | Y | Y | U | Y | 10 | **Low** |
| Garcia 2016  [13] | 149 | Retrospective cohort | Y | Y | Y | U | U | Y | Y | Y | U | U | Y | 7 | **Moderate** |
| Hari Gopal 2023  [91] | 57 | Retrospective cohort | Y | Y | Y | U | U | Y | Y | Y | U | U | Y | 7 | **Moderate** |
| Kim 2015  [59] | 126 | Retrospective cohort | Y | Y | Y | U | U | Y | Y | Y | U | U | Y | 7 | **Moderate** |
| Kim-Campbell 2020 [79] | 34 | Prospective cohort | Y | Y | Y | U | U | Y | U | Y | U | U | U | 5 | **Moderate** |
| Kulyabin 2020a  [66] | 121 | Retrospective cohort | Y | Y | Y | U | U | Y | Y | Y | U | U | Y | 7 | **Moderate** |
| Kulyabin 2020b  [71] | 45 | Prospective randomized (pilot) | Y | Y | Y | U | U | Y | Y | Y | U | U | Y | 7 | **Moderate** |
| Kumar 2014  [57] | 208 | Retrospective cohort | Y | Y | Y | U | U | Y | Y | Y | U | U | Y | 7 | **Moderate** |
| Kuraim 2018  [72] | 565 | Prospective cohort | Y | Y | Y | U | U | Y | Y | Y | U | U | Y | 7 | **Moderate** |
| Lex 2016  [63] | 1520 | Prospective cohort  (secondary analysis) | Y | Y | Y | Y | Y | Y | Y | Y | U | U | Y | 9 | **Low** |
| Luo 2020  [46] | 504 | Retrospective case control | Y | Y | Y | Y | Y | Y | Y | Y | U | U | Y | 9 | **Low** |
| Miletic 2015  [36] | 222 | Retrospective cohort | Y | Y | Y | Y | Y | Y | Y | Y | U | U | Y | 9 | **Low** |
| Murin 2020  [78] | 615 | Retrospective cohort | Y | Y | Y | Y | Y | Y | Y | Y | U | U | Y | 9 | **Low** |
| Odek 2016  [61] | 99 | Prospective cohort | Y | Y | Y | U | U | Y | Y | Y | U | U | Y | 7 | **Moderate** |
| Odek 2018  [67] | 126 | Retrospective cohort | Y | Y | Y | U | U | Y | Y | Y | U | U | Y | 7 | **Moderate** |
| Parmar 2017  [65] | 135 | Prospective cohort | Y | Y | Y | U | U | Y | Y | Y | U | U | Y | 7 | **Moderate** |
| Pérez-Navero 2019  [74] | 117 | Prospective cohort | Y | Y | Y | Y | Y | Y | Y | Y | U | U | Y | 9 | **Low** |
| Raatz 2019  [75] | 745 | Retrospective cohort | Y | Y | Y | U | U | Y | Y | Y | U | U | Y | 7 | **Moderate** |
| Radbill 2022  [84] | 1250 | Prospective cohort | Y | Y | Y | Y | Y | Y | Y | Y | U | U | Y | 8 | **Low** |
| Sanil 2013  [7] | 51 | Retrospective cohort | Y | Y | Y | Y | Y | Y | Y | Y | U | U | Y | 9 | **Low** |
| Scherer 2016  [60] | 164 | Prospective cohort | Y | Y | Y | Y | Y | Y | Y | Y | U | U | Y | 9 | **Low** |
| Schroeder 2018  [53] | 34 | Retrospective cohort | Y | Y | Y | U | U | Y | Y | Y | U | U | Y | 7 | **Moderate** |
| Shaath 2020  [15] | 96 | Case-control | N | U | Y | U | U | N | Y | U | U | U | Y | 3 | **High** |
| Siehr 2016  [62] | 32 | Retrospective cohort | Y | Y | Y | U | U | Y | Y | Y | Y | Y | Y | 9 | **Low** |
| Song 2022  [85] | 282 | Retrospective cohort | Y | Y | Y | U | U | Y | Y | Y | U | U | Y | 7 | **Moderate** |
| SooHoo 2018  [69] | 95 | Retrospective cohort | Y | Y | Y | U | U | Y | Y | Y | U | U | Y | 7 | **Moderate** |
| Sun 2022  [33] | 401 | Retrospective cohort | Y | Y | Y | Y | Y | Y | Y | Y | U | U | Y | 9 | **Low** |
| Tabbutt 2019  [76] | 8543 | Observational analysis | Y | Y | Y | Y | Y | Y | Y | Y | N | U | Y | 9 | **Low** |
| Tadros 2020  [77] | 104 | Retrospective cohort | Y | Y | Y | Y | Y | Y | Y | Y | U | U | Y | 9 | **Low** |
| Talwar 2018  [70] | 215 | Retrospective cohort | Y | Y | Y | U | U | Y | Y | Y | Y | Y | Y | 9 | **Low** |
| Yokota 2022  [83] | 70 | Retrospective matched cohort | Y | Y | Y | U | U | Y | Y | Y | U | U | Y | 7 | **Moderate** |
| Zhang 2018  [47] | 71 | Retrospective case-control | Y | Y | Y | U | U | Y | Y | U | U | U | Y | 6 | **Moderate** |
| Zhang 2020  [80] | 451 | Retrospective cohort | Y | Y | Y | Y | Y | Y | Y | Y | U | U | Y | 9 | **Low** |
| **SECTION 3 — ADULT SHOCK (SEPTIC AND NON-SEPTIC) (n = 6)** | | | | | | | | | | | | | | | |
| Kara 2019a  [92] | 102 | Retrospective cohort | Y | Y | Y | U | U | U | Y | Y | U | U | Y | 6 | **Moderate** |
| Kara 2019b  [93] | 392 | Retrospective cohort | Y | Y | Y | Y | Y | U | Y | Y | U | U | Y | 8 | **Moderate** |
| Li 2024  [40] | 4229 | Retrospective cohort | Y | Y | Y | Y | Y | U | Y | Y | U | U | Y | 8 | **Moderate** |
| Ning 2023  [18] | 8887 | Retrospective cohort | Y | Y | Y | Y | Y | U | Y | Y | U | U | Y | 8 | **Low** |
| Pölkki 2023  [94] | 8079 | Retrospective cohort | Y | N | Y | Y | Y | U | Y | Y | U | U | Y | 7 | **Moderate** |
| Song 2021  [49] | 910 | Retrospective cohort | Y | Y | Y | Y | Y | U | Y | Y | U | U | Y | 8 | **Moderate** |
| **SECTION 4 — ADULT CARDIAC SURGERY / MIXED (n = 26)** | | | | | | | | | | | | | | | |
| Barge-Caballero 2015 [5] | 390 | Retrospective cohort | Y | Y | Y | U | U | Y | Y | U | U | U | Y | 6 | **Moderate** |
| Baysal 2021  [101] | 290 | Prospective cohort | Y | Y | Y | U | U | Y | Y | U | U | U | Y | 6 | **Moderate** |
| Carmona 2020  [98] | 68 | Retrospective cohort | Y | Y | Y | U | U | Y | Y | U | U | U | Y |  | **Moderate** |
| Caruso 2024  [29] | 75 | Retrospective multicenter (PSM) | U | Y | Y | U | U | Y | Y | U | U | U | Y | 5 | **Moderate** |
| Chen 2020  [22] | 121 | Retrospective cohort | Y | Y | Y | U | U | Y | Y | Y | U | U | Y | 7 | **Moderate** |
| Han 2019  [96] | 418 | Retrospective cohort | Y | Y | Y | U | U | Y | Y | U | U | U | Y | 6 | **Moderate** |
| Han 2021  [99] | 90 | Retrospective cohort | Y | Y | Y | U | U | Y | Y | U | U | U | Y | 6 | **Moderate** |
| Hou 2021  [100] | 1935 | Retrospective cohort | Y | Y | Y | U | U | Y | Y | U | U | U | Y | 6 | **Moderate** |
| Jiang 2022  [103] | 84 | Retrospective cohort | Y | Y | Y | U | U | Y | Y | U | U | U | Y | 6 | **Moderate** |
| Keles 2024  [89] | 221 | Retrospective cohort | Y | Y | Y | U | U | Y | Y | Y | U | U | Y | 7 | **Moderate** |
| Knight 2022  [104] | 245 | Retrospective cohort | Y | Y | Y | U | U | Y | Y | U | U | U | Y | 6 | **Moderate** |
| Koponen 2019  [34] | 3213 | Retrospective cohort | U | Y | Y | U | U | Y | Y | Y | U | U | Y | 6 | **Moderate** |
| Kwon 2022  [102] | 2149 | Retrospective cohort | Y | Y | Y | U | U | Y | Y | Y | U | U | Y | 7 | **Moderate** |
| Lim 2017  [14] | 160 | Retrospective case-control | N | U | Y | U | U | N | Y | U | U | U | Y | 3 | **High** |
| Liu 2018  [95] | 112 | Retrospective cohort | Y | Y | Y | U | U | Y | Y | U | U | U | Y | 6 | **Moderate** |
| Liu 2024  [88] | 250 | Retrospective cohort | Y | Y | Y | Y | Y | Y | Y | Y | U | U | Y | 9 | **Low** |
| Loforte 2021  [106] | 499 | Retrospective cohort | Y | Y | Y | U | U | U | Y | U | Y | U | Y | 6 | **Moderate** |
| Poterucha 2019  [21] | 243 | Retrospective cohort | Y | Y | Y | Y | Y | Y | Y | Y | U | U | Y | 9 | **Low** |
| Schnackenburg 2025 [86] | 334 | Retrospective cohort | Y | Y | Y | U | U | Y | Y | Y | U | U | Y | 7 | **Moderate** |
| Singh 2022  [105] | 687 | Retrospective cohort | Y | Y | Y | U | U | Y | Y | U | U | U | Y | 6 | **Moderate** |
| Sunavsky 2018  [97] | 447 | Retrospective cohort | Y | U | Y | U | U | Y | Y | U | U | U | Y | 5 | **Moderate** |
| Tohme 2023  [48] | 151 | Retrospective cohort | Y | Y | Y | U | U | Y | Y | U | U | U | Y | 6 | **Moderate** |
| Vandenberghe 2022 [17] | 3415 | Retrospective cohort | Y | Y | Y | U | U | Y | Y | Y | U | U | Y | 7 | **Moderate** |
| Xiong 2024  [50] | 325 | Retrospective cohort | Y | Y | Y | U | U | Y | Y | U | Y | Y | Y | 8 | **Low** |
| Yamazaki 2018  [30] | 129 | Retrospective cohort | Y | Y | Y | U | U | Y | Y | U | U | U | Y | 6 | **Moderate** |
| Zhao 2024  [87] | 409 | Retrospective cohort | Y | Y | Y | U | U | Y | Y | Y | U | U | Y | 7 | **Moderate** |
| **SECTION 5 — ADULT CARDIOGENIC SHOCK / VA-ECMO (n = 8)** | | | | | | | | | | | | | | | |
| Hu 2024  [108] | 1742 | Retrospective cohort | Y | Y | Y | U | U | Y | Y | Y | U | U | Y | 7 | **Moderate** |
| Hyun 2021  [42] | 160 | Retrospective cohort | Y | Y | Y | Y | Y | Y | Y | Y | Y | Y | Y | 11 | **Low** |
| Na 2019  [16] | 493 | Retrospective cohort | U | Y | Y | U | U | U | Y | U | U | U | Y | 4 | **High** |
| Ning 2026  [19] | 3170 | Retrospective cohort | Y | Y | Y | Y | Y | Y | Y | Y | U | U | Y | 9 | **Low** |
| Sandrio 2022  [43] | 221 | Retrospective cohort | Y | Y | Y | U | U | Y | Y | Y | U | U | Y | 7 | **Moderate** |
| Tian 2024  [41] | 222 | Retrospective cohort | Y | Y | Y | U | U | Y | Y | Y | U | U | Y | 7 | **Moderate** |
| Weeks 2023  [107] | 265 | Retrospective cohort | Y | Y | Y | Y | U | Y | Y | Y | U | U | Y | 6 | **Moderate** |
| Zhang 2024  [51] | 126 | Retrospective cohort | Y | Y | Y | U | U | Y | Y | Y | U | U | Y | 7 | **Moderate** |
